# A Question of Ethics

**Published:** 1889-01

**Authors:** 


### A QUESTION OF ETHICS.—ANSWER WANTED.

*Editor Daniel's Texas Medical Journal:*

Will you please publish this in your JOURNAL, and answer as to the ethics or etiquette, or if either or all parties were wrong?

Dr. A. was called to see a case of passive epixtaxis about 11, a. m., in a family in which he had been practicing for years: (case a very stout man, though just over a slight attack of pneumonia, in which Dr. A. had attended him,) used some of the ordinary remedies, and left, after bleeding had ceased; called by about 2 o'clock, and there was found slight bleeding, dripping; plugged up the anterior nares with absorbent cotton after insufflation of hot water, when bleeding ceased, and Dr. A. left; called again about 5:30, p. m., and found the room full of people and Dr. C. there also, who told Dr. A. that Dr. B. had just left, and he and Dr. B. had plugged the nose posteriously with struice ] and cotton, and one nostril anteriorly; Dr. C. said the bleeding was not at all alarming; he remarked "it was bleeding some." When Dr. A. entered, seeing Dr. C., he, with astonishment remarked, "you here!" when he replied as above, and the wife of the sick man said: "I did not want to see my husband die, and sent for the other doctors." Dr. A. was just as near, and accessible as either of the others; Dr. A. left, without any instructions to the patient; Dr. C. left at same time, remarking to a lady who had asked "if patient needed some milk," "we need it worse than he."

Next morning they sent foı Dr. A., who refused to go; all the doctors are members of district association.

[If this could be classed as "an emergency" Dr. C. should have stated to Dr. A. that he had so understood it, told him what had been done, and turned the case over to him and retired; and Dr. A. should have continued in charge, (with the consent of the family, of course.) If the family wished to change the physician Dr. A. should have been notified before Drs. C. and B. could take the case.

Under the circumstances, and from the above presentation of the case we think Drs. C. and B. were wrong in taking the case if they knew it was in the hands of Dr. A., and on this point they should have been informed; and Dr. A. was right in refusing to be made use of next day.—ED.]

